# The effects of WeChat follow-up management to improve the parents’ mental status and the quality of life of premature newborns with patent ductus arteriosus

**DOI:** 10.1186/s13019-021-01617-5

**Published:** 2021-08-21

**Authors:** Bin Yang, Jian-Feng Liu, Wen-Peng Xie, Hua Cao, Qiang Chen

**Affiliations:** 1 Department of Neonatology, Fujian Maternity and Child Health Hospital, Affiliated Hospital of Fujian Medical University, Fuzhou, China; 2 Department of Cardiac Surgery, Fujian Branch of Shanghai Children’s Medical Center, Fuzhou, China; 3 Fujian Children’s Hospital, Fuzhou, China; 4 Fujian Maternity and Child Health Hospital, Affiliated Hospital of Fujian Medical University, Fuzhou, China; 5 Fujian Key Laboratory of Women and Children’s Critical Diseases Research, Fujian Maternity and Child Health Hospital, Fuzhou, China

**Keywords:** Follow-up, PDA, Social media

## Abstract

**Objective:**

This study aimed to explore the effect of WeChat follow-up management on improving the parents’ mental status and the quality of life of premature newborns with patent ductus arteriosus (PDA) after discharge.

**Methods:**

Participants were randomly divided into an intervention group and a control group. WeChat was used in the intervention group for the postdischarge follow-up management, while the control group was managed traditionally. The psychological status and quality of life of the parents of the two groups were analyzed and compared.

**Results:**

The SAS and SDS scores in the intervention group at three months after discharge were significantly better than those at discharge, but there was no significant change in the control group. During the three-month follow-up, the SAS and SDS scores in the intervention group were significantly better than those in the control group. The WHOQOL-BREF scale scores in the intervention group were significantly better than those in the control group in physiology, psychology, social relations, and the environment. The total quality of life score in the intervention group was also significantly better than that in the control group. All patients in the intervention group were followed up as appropriate, while five patients in the control group were lost to follow-up. The incidence of pneumonia and feeding intolerance in the intervention group were significantly lower than those in the control group.

**Conclusion:**

The application of WeChat in the follow-up management of premature infants with PDA could alleviate parents’ anxiety and depression in taking care of their children at home and can improve their quality of life.

## Introduction

Unlike persistent ductus arteriosus, patent ductus arteriosus (PDA) should not be considered a congenital heart disease (CHD) but a cardiovascular condition resulting from its later closure in premature newborns. It has been reported that approximately 10% of premature infants with a gestational age of 30–37 weeks have PDA, and approximately 80% of premature infants with a gestational age of 25–28 weeks have PDA. Approximately 90% of premature infants less than 24 weeks of gestational age have PDA [1, 2]. With the development of medical research and due to the high rate of spontaneous closure of a PDA in premature infants, surgical trauma, and side effects of drug treatment, more practitioners are opting for just observing PDA with appropriate follow-up [3–5]. However, the spontaneous closure of PDA in premature infants is often later than that in full-term infants. The persistence of PDA will adversely affect the health of premature infants and make them more vulnerable [6].

Due to the immature organs of premature infants and the effects of abnormal PDA hemodynamics, these premature infants are prone to a variety of adverse events in the process of home care, so they need professional and meticulous care [7]. Due to the lack of professional medical staff support after discharge, many families feel pressured to take care of premature infants with PDA. They may be busy taking care of their children all day and can worry about the occurrence of adverse events and their child’s illness progressing, resulting in their poor mental status and lower quality of life. Some studies have shown that applying the WeChat platform for continuous follow-up management and medical support for discharged patients can effectively reduce patients’ family care pressure and anxiety and improve their quality of life [8–10]. Currently, there are no reports in the literature regarding the application of WeChat in the follow-up management of premature infants with PDA after discharge. This study conducted a prospective randomized controlled study to evaluate the effect of WeChat follow-up management on improving the parents’ mental status and the quality of life of premature newborns with patent ductus arteriosus (PDA) after discharge.

## Methods

The present study was approved by the ethics committee of the Fujian Maternity and Child Health Hospital and adhered to the Declaration of Helsinki’s tenets. Additionally, all parents signed the consent form before participating in the study.

### Study design

This study was a prospective randomized controlled study conducted by a provincial hospital in China. The clinical and family data of 94 premature infants with PDA diagnosed and treated in our hospital were collected from June 2019 to February 2020. The inclusion criteria were as follows: 1. premature infants with PDA; 2. parents were the primary caregivers; and 3. parents had smartphones and were proficient in using the WeChat platform. The exclusion criteria were as follows: 1. patients complicated with other congenital heart malformations; 2. severe conditions requiring an emergency operation or long-term drug treatment; 3. patients complicated with other neonatal diseases; and 4. parents refusing to participate in the study or follow-up program.

According to the differences between the two groups of patients in the presurvey results, we assumed that the difference between the two independent populations was 10%, α = 0.05, β = 0.2, and we determined that 42 participants were needed in each group. We assumed a 10% loss of follow-up rate, and the total sample size required was 94 cases (47 cases in each group). According to the computer-generated random numbers method, the researchers randomly divided the eligible patients into the intervention group (the parents would use WeChat with access to medical caregivers) and the control group (the parents received a pamphlet and had access to medical caregivers).

### Intervention methods

The patients in the intervention group used the WeChat platform for follow-up and health education. In the intervention group, medical staff who participated in the study registered a WeChat official account specifically for the follow-up management, asked all of the parents to join this official account, and instructed the parents to receive and send messages by WeChat. The content of the WeChat platform in the intervention group mainly included two modules: the education module and question module. The education module included nursing and feeding information for premature infants with PDA, knowledge related to PDA and related adverse events, parents’ psychological counseling, etc. Parents could read this information anytime and anywhere. In the question and answer module, the team’s medical staff was on duty every day and was online from 18:00 to 22:00 to carefully go over the parents’ concerns, remind and supervise all patients to have regular outpatient reexaminations, and remind the parents what the time the operation would be. The staff also actively guided parents to communicate and share their care experience with other parents on the platform. For parents who were pessimistic, anxious, or depressed, the researchers provided individual psychological counseling and support through the WeChat platform.

The parents of the patients in the control group received the education pamphlet when they were discharged from the hospital, which contained the same educational content and follow-up time as in the education module of the intervention group and reminded the parents to go to the hospital immediately if there was an emergency.

### Evaluation tool

Zung’s self-rating anxiety scale (SAS) is widely used in the clinical evaluation of anxiety, and it has good psychometric properties [11]. The SAS is a self-reported scale that contains 20 items covering various anxiety symptoms. Responses are given on a 4-point scale, ranging from 1 (none, or a little of the time) to 4 (most, or all of the time). Participants are instructed to base their answers on their experiences over the last week. Items include both negative and positive experiences, with the latter being reverse scored. Raw scale scores for the SAS ranged from 20 to 80. Score description: < 50 indicates normal, 50–59 indicates mild anxiety, 60–69 indicates moderate anxiety, and ≥ 70 indicates severe anxiety.

Zung’s self-rating depression scale (SDS) is widely used in the clinical evaluation of depression; it has good psychometric properties [12]. The SDS consists of 20 self-report items identified in factor analytic studies of the syndrome of depression. Factors include psychological and physiological symptoms; 10 express negatives, and 10 express positives. Respondents rated each item according to how it applied to them within the past week using a 4-point scale ranging from 1 (none, or a little of the time) to 4 (most, or all of the time). Total raw scores range from 20 to 80. Score description: 50–59 indicates mild depression, 60–69 indicates moderate depression, and ≥ 70 indicates severe depression.

The WHOQOL-BREF scale was developed based on the WHOQOL-100 [13]. The scale retains the original scale’s comprehensiveness, and the scores in each field are positively correlated with those in the corresponding fields of the original scale (the correlation coefficient is between 0.89 and 0.95) [14]. The scale consists of 26 items, including the physiological field, psychological field, social relations field, and environmental field. Item 1 and item 2 are independent topics, and the total score of item 1 plus item 2 is used as an overall index to evaluate the quality of life. Each item in the scale is designed as a scale of 1–5, corresponding to 1–5 points, of which three items (3, 4, 26) are the reverse scoring structure, and 1–5 grades correspond to 5–1 points. The higher the total score, the higher the quality of life. The scale has excellent reliability and validity.

### Data collection

The parents of all of the patients completed the SAS scale and the SDS scale when they were discharged from the hospital and then completed the SAS scale, the SDS scale, and the WHOQOL-BREF scale again when they were reevaluated during the three months of follow-up. Data related to adverse events (including pneumonia, feeding intolerance, cholestasis, liver insufficiency, renal insufficiency, necrotizing enterocolitis, asphyxia, and death) during the 3-month follow-up were also collected.

### Statistical analysis

All statistical analyses were conducted with SPSS 23.0 software. Continuous data are expressed as the mean ± standard deviation. A routine distribution test was performed for all continuous data, and the data were confirmed to be normally distributed. Clinical parameters between the two groups were compared with the independent samples t-test. The χ^2^ or Fisher’s exact test was used for categorical variables. A P-value of < 0.05 was defined as statistically significant.

## Results

The general data of all patients and families are shown in Table 1, and there was no significant difference between them. There was also no significant difference in the SAS and SDS scores of the parents between the two groups at discharge. These results showed that the patients and families were homogeneous and comparable between the two groups (Table 1).

**Table 1:** Comparison of clinical characteristics between the two groups

	Intervention group	Control group	P value
Gestational age (weeks)	34.5 ± 2.1	33.4 ± 2.5	0.542
Ductus arteriosus size (mm)	2.8 ± 0.5	2.9 ± 0.6	0.745
Pulmonary artery pressure (mmHg)	43.2 ± 15.4	45.5 ± 14.5	0.388
Weight (kg)	2.0 ± 0.8	2.1 ± 07	0.861
Height (cm)	26.3 ± 7.1	28.2 ± 6.2	0.402
Parents’ educational level			
Below senior high school	6	7	
High school	15	16	
Junior college	18	17	0.655
Bachelor degree or above	8	7	
Living environment			
Rural areas	30	32	0.663
City	17	15	

The SAS and SDS scores at the three-month follow-up in the intervention group were significantly better than those at discharge, but there was no significant difference in the control group. During the three-month follow-up, the intervention group’s SAS and SDS scores were significantly better than those in the control group (P < 0.05). Through the WeChat intervention, the anxiety and depression of parents were significantly improved (Table 2).

**Table 2:** Comparison of the scores of SAS and SDS scale of the parents between the two groups

	Intervention group	Control group	P_1_ value
At discharge			
SAS score	65.2 ± 14.2	66.3 ± 16.3	0.512
SDS score	62.5 ± 11.3	63.3 ± 12.6	0.496
Three-month follow-up			
SAS score	48.2 ± 9.7	60.8 ± 15.2	0.019
SDS score	50.1 ± 10.6	61.5 ± 13.4	0.028
P_2_ value	0.013	0.236	
P_3_ value	0.021	0.428	

P_1_ value indicates the result of comparison between intervention group and control group

P_2_ value indicates the result of comparison of SAS score between 3-months follow-up and at discharge time

P_3_ value indicates the result of comparison of SDS score between 3-months follow-up and at discharge time

The WHOQOL-BREF scale results showed that scores in the physiological field, psychological field, social relationship field, and environmental field in the intervention group were significantly better than those in the control group during the three-month follow-up (P < 0.05). The total quality of life score in the intervention group was also significantly better than that in the control group (P < 0.05) (Table 3).

**Table 3:** Comparison of the scores of WHOQOL-BREF scale of the parents between the parents of the two groups at 3 months follow up

	Intervention group	Control group	P value
Physiological field	11.8 ± 1.5	9.6 ± 1.2	0.037
Psychological field	13.6 ± 1.9	10.4 ± 1.6	0.023
Social relations field	14.0 ± 2.1	9.9 ± 1.1	0.021
Environmental field	13.7 ± 2.0	10.2 ± 1.8	0.032
Total score of quality of life	61.2 ± 3.4	45.8 ± 3.1	0.013

All patients in the intervention group were followed up, but five patients in the control group were lost to follow-up; the difference was statistically significant (P < 0.05). During the follow-up period, the incidence of pneumonia and feeding intolerance in the intervention group was significantly lower than that in the control group (P < 0.05). There was no significant difference in the incidence of jaundice, cholestasis, or necrotizing enterocolitis between the two groups. There were no other related adverse events, such as renal insufficiency, asphyxia, or death, in either group (Table 4).

**Table 4:** Comparison of adverse events between the two groups during 3 months follow up

	Intervention group	Control group	P value
Lost to follow up	0	5	0.022
Pneumonia	2	8	0.027
Feeding intolerance	1	6	0.033
Jaundice	3	3	0.887
Cholestasis	2	3	0.555
Necrotizing enterocolitis	0	1	–
Renal insufficiency	0	0	–
Asphyxia	0	0	–
Death	0	0	–

## Discussion

Due to the poor function of various organs, premature infants are prone to many complications while getting home care [15, 16]. Premature infants with PDA may have serious health issues, leading to more adverse events and emergencies, and they can be more difficult to feed [7]. In China, advanced medical resources and medical support are usually provided during the period of hospitalization. When these patients are discharged from the hospital, contact between the patients and medical staff is often interrupted. It is inconvenient and difficult to seek professional medical support for parents that live in remote areas. During the home care process, the parents are concerned about adverse events and emergencies and can be in a state of mental tension or can even be distraught, and their quality of life can decline from the anxiety With the improvement of people’s living standards, people’s requirements for quality of life are increasing. Studies have shown that social support is positively correlated with caregivers’ ability and quality of life [17]. To improve parents’ psychological status and their quality of life, we applied the WeChat platform to the follow-up management of premature infants with PDA after discharge and continuously provided medical support to parents.

Feeding intolerance and pneumonia are the most common adverse events in premature infants with PDA [18]. A systematic review of 36 reports that were published during 2014–2018 showed that the patients who received WeChat follow-up had a lower risk of PICC-related complications and received better home care [19]. A study from Dong et al. showed that health education for diabetic patients via the WeChat platform could improve glycemic control and reduce complications [20]. This study also proved that through the WeChat platform’s medical support, the parents in the intervention group could learn professional knowledge and consult the medical staff at any time, which could improve their care and nursing ability and reduce adverse events while patients are receiving home care. Therefore, the incidence of feeding intolerance and pneumonia in the intervention group was significantly lower than that in the control group. Through the WeChat platform, health professionals could also understand parents’ psychological state, give guidance and support, encourage them to adopt complementary coping styles, alleviate negative psychology, and eliminate worries. At the same time, health professionals could also encourage the family members in the WeChat group to communicate with each other and share successful cases and experiences so that those parents could be supported by the group, which can enhance their confidence and hope. All of these factors were conducive to reducing the anxiety and depression of parents.

Many studies have shown that the psychological pressure of home care mainly came from the lack of understanding of the disease and the lack of professional medical support, causing these caregivers to develop chronic tension and anxiety, resulting in a decline in their quality of life [21, 22]. Study of Okhovat et al. showed that providing continuous medical support to the children discharged from the pediatric surgical unit could ease the mother’s anxiety [23]. Rahim et al. showed that providing continuous medical support to hemodialysis patients could improve the quality of life of spouses [24]. In this study, the health professionals could extend the hospital high-quality professional health care service to the family through the WeChat platform after discharge, which could effectively improve the level of care of the patients, alleviate parents’ anxiety and depression, and improve the quality of life of the parents.

There were some limitations to this paper. First, due to Internet instability, especially in rural areas of China, many families could not easily access WeChat. Second, the sample size of the study was relatively limited, and the follow-up time was short. Third, most of the indicators adopted in this study were subjective indicators, which may have introduced selection bias and might have affected the accuracy of the results. Our future studies would further confirm these findings in a larger cohort using a longer follow-up period.

## Conclusion

The application of WeChat to the follow-up management of premature infants with PDA can provide a timely understanding of the patients’ physical status and provide significant medical support. This intervention also helps alleviate parents’ anxiety and depression in taking care of their children at home to improve their quality of life.

## Data Availability

The data sets used and/or analyzed during the current study are available from the first author or the corresponding author on reasonable request.

## Abbreviations

PDA: Patent ductus arteriosus
CHD: Congenital heart disease
